# Induction of lung lesions in Wistar rats by 4-(methylnitrosamino)-1-(3-pyridyl)-1-butanone and its inhibition by aspirin and phenethyl isothiocyanate

**DOI:** 10.1186/1471-2407-7-90

**Published:** 2007-05-29

**Authors:** Bo Ye, Yu-Xia Zhang, Fei Yang, Hong-Lei Chen, Dong Xia, Ming-Qiu Liu, Bai-Tang Lai

**Affiliations:** 1 Pathology Department, Basic Medical School, Wuhan University, Dong-Hu Road, Wuhan, P.R. China; 2 Beijing Tuberculosis and Thoracic Tumor Research Institute, Beijing 101149, P.R. China

## Abstract

**Background:**

The development of effective chemopreventive agents against cigarette smoke-induced lung cancer could be greatly facilitated by suitable laboratory animal models, such as animals treated with the tobacco-specific lung carcinogen 4-(methylnitrosamino)-1-(3-pyridyl)-1-butanone (NNK). In the current study, we established a novel lung cancer model in Wistar rats treated with NNK. Using this model, we assessed the effects of two chemopreventive agents, aspirin and phenethyl isothiocyanate (PEITC), on tumor progression.

**Methods:**

First, rats were treated with a single-dose of NNK by intratracheal instillation; control rats received iodized oil. The animals were then sacrificed on the indicated day after drug administration and examined for tumors in the target organs. PCNA, p63 and COX-2 expression were analyzed in the preneoplastic lung lesions. Second, rats were treated with a single-dose of NNK (25 mg/kg body weight) in the absence or presence of aspirin and/or PEITC in the daily diet. The control group received only the vehicle in the regular diet. The animals were sacrificed on day 91 after bronchial instillation of NNK. Lungs were collected and processed for histopathological and immunohistochemical assays.

**Results:**

NNK induced preneoplastic lesions in lungs, including 33.3% alveolar hyperplasia and 55.6% alveolar atypical dysplasia. COX-2 expression increased similarly in alveolar hyperplasia and alveolar atypical dysplasia, while PCNA expression increased more significantly in the latter than the former. No p63 expression was detected in the preneoplastic lesions. In the second study, the incidences of alveolar atypical dysplasia were reduced to 10%, 10% and 0%, respectively, in the aspirin, PEITC and aspirin and PEITC groups, compared with 62.5% in the carcinogen-treated control group. COX-2 expression decreased after dietary aspirin or aspirin and PEITC treatment. PCNA expression was significantly reduced in the aspirin and PEITC group.

**Conclusion:**

(1) A single dose of 25 mg/kg body weight NNK by intratracheal instillation is sufficient to induce preneoplastic lesions in Wistar rat lungs. (2) COX-2 takes part in NNK-induced tumorigenesis but is not involved in proliferation. (3) Aspirin and PEITC have protective effects in the early stages of tumor progression initiated by NNK.

## Background

Lung cancer is strongly associated with cigarette smoking [1]. It is the single best-documented risk factor for all lung cancer types [2]. Each cigarette contains a mixture of carcinogens, including the tobacco-specific nitrosamine NNK and a small dose of polycyclic aromatic hydrocarbons (PAHs) among others, along with tumor promoters and co-carcinogens [3,4]. The complex of PAHs and the tobacco-specific nitrosamine NNK in cigarette smoke is the mixture that is most likely to be involved in the induction of human lung cancer.

It is suggested that NNK is strongly related to human pulmonary adenocarcinoma [2,5,6]. An etiological role of NNK in the induction of human lung cancer is supported by evidence showing that NNK is activated to form a DNA-damaging agent in human lung tissue [7], and that the lung is an important target organ for NNK-induced carcinogenesis in rodents. NNK induces lung adenocarcinoma in mice, rats and Syrian golden hamsters, independently of administration route [8-11]. Although these models are widely used to explore the genetic mechanism(s) of pulmonary neoplasia and its chemoprevention, some weaknesses cannot be ignored. First, the animals are usually given carcinogens by intraperitoneal (i.p.) or subcutaneous injection (s.c.) or oral administration, and administration takes a long time. Second, neoplasia can be found in other organs besides lung, e.g. nasal cavity, liver, esophagus and pancreas. Third, a high incidence of spontaneous tumors including pulmonary neoplasia has been observed in some special strains used in this model, e.g. A/J mouse.

In the present work, we show that a single dose of NNK by intratracheal instillation induces preneoplastic lesions in Wistar rat lungs. This is the first time, to our knowledge, that early preneoplastic lesions have been reported in NNK-treated Wistar rats. The advantages and limitations of the model in chemoprevention studies are discussed and we also suggest that two chemopreventive agents, PEITC and aspirin, protect the rats against NNK-induced tumorigenesis during the early stages of tumor development. Understanding the different early steps of lung tumorigenesis caused by NNK might facilitate the development of more effective strategies for the prevention, early diagnosis and treatment of lung cancer.

## Methods

### Animals

Female Wister rats, 6–7 weeks, 200 ± 10 g, were purchased from the animal center of Hubei College of Traditional Chinese Medicine. The animals were housed in plastic cages under standard laboratory condition (25°C; 40% relative humidity; 12 h dark/light cycle) with food and water. We received approval for the production and use of laboratory animals (rodent, rabbit, dog and monkey) from Hubei Province Department of Science and Technology, license No. SYXK () – 2003 – 0013.

### Chemicals

NNK was purchased from Chemsyn Science Laboratories. PEITC and aspirin were from Sigma-Aldrich.

### Tumor induction

NNK was dissolved in iodized oil at a stock concentration of 50 mg/ml. The animals were randomly assigned to 2 groups. Group 1, comprising 18 animals, were instilled with NNK into the left lobe using the method previously established [12]. In brief: after the animal was anesthetized, it was hung on a slanted surgical board and its vocal cord was exposed. Using a blunt ZY-type 12-gauge needle, NNK was instilled into the left lower lobe at a dose of 25 mg/kg body weight. Group 2, comprising 10 animals, were instilled with 0.1 ml iodized oil into the left lung as control. After instillation, the rats were given streptomycin (250 mg/kg body weight) and penicillin (100000 U/kg body weight) by muscle injection for 7 days to preclude infections.

### DSA of X-ray photography

Every animal was X-rayed immediately after instillation to identify the location of the iodized oil, and again after 92 days to observe changes in the left lobe.

### Histopathology

All animals that died during the study or were sacrificed under ether anesthesia received a complete necropsy and histopathological examination. Lungs were perfused intratracheally with 10% neutral buffered formalin (NBF) and immersed in 10% NFB for fixation. Major tissues were fixed and preserved in 10% NFB, processed and trimmed, embedded in paraffin, sectioned at a thickness of 3–4 μm, and stained with hematoxylin and eosin for microscopic examination. The following tissues were examined microscopically for gross lesions and tissue masses: bone and marrow, heart, large intestine, small intestine, kidney, liver, lung and associated lymph nodes, spleen, stomach, nasal cavity. Three transverse step sections were prepared to observe tumor development in the left lung of each rat. The sections from group 1, which received NNK, were divided into alveolar hyperplasia, alveolar atypical dysplasia, adenoma and adenocarcinoma. The following criteria for diagnostic evaluation were used: alveolar hyperplasia – alveolar structure of lung is present, proliferation of cells obliterates normal alveolar space, and the proliferated cells are homogeneous and slightly enlarged without cytological atypia. Alveolar atypical dysplasia – alveolar structure of lung is largely present, and the proliferated cells are larger and show enlarged nuclei, hyperchromasia and nuclear-cytoplasmic reversal. Adenoma – alveolar structure is largely absent and replaced by proliferated cells with or without cytological atypia. Adenocarcinoma – tumor composed of glandular, papillary, or solid masses with cytological atypia. Preneoplastic lesions include alveolar hyperplasia and atypical dysplasia.

### Transmission electron microscopy

To identify the origin of the proliferated cells in the group treated with NNK (group 1), small pieces of specimens from the left lung were fixed in 5% phosphate buffered glutaraldehyde for 2 hours, then postfixed in 1% osmium tetroxide, dehydrated in a graded series of ethanol and acetone and embedded in epoxy resin 618. Ultrathin sections were stained with uranyl acetate and lead citrate and examined with a Hitachi H-600 electron microscope.

### Immunohistochemistry

Antibodies against CK (AE1/AE3), PCNA, p63, COX-2 (rabbit polyclonal) were purchased from NeoMarkers (Lab Vision, Fremont, CA). IHC staining was performed using a modified avidin-biotin complex method described by Kobayashi [13]. Sections of formalin-fixed and paraffin-embedded tissue samples from gross lesions of the lung were deparaffinized in xylene and hydrated through a graded alcohol series before being microwaved in citrate buffer (pH 6) for 10 minutes, and the slides were then allowed to cool at room temperature for 30 minutes. Endogenous peroxidase was blocked with 3% hydrogen peroxide for 15 minutes, and the tissues were incubated with normal blocking goat serum. Subsequently, the slides were incubated with primary antibody (dilution, 1:200) overnight at 4°C, washed, stained with biotinylated second antibody for 30 minutes, incubated with strepavidin horseradish peroxidase enzyme at room temperature for 20 minutes, then treated with 3', 3-diaminobenzidine chromogen (DAB) for 3 minutes at room temperature and counterstained with hematoxylin.

PCNA immunostaining [14] was scored by counting at least 500 cells in representative high power fields. Every stained nucleus was considered positive, irrespective of intensity. In cases where staining was heterogeneous in the section, the fields examined included those with the highest and those with the lowest percentages of stained cells. The percentage of positive stained cells was recorded as the PCNA labeling index (LI). In preneoplastic lesions, p63 was scored on the following basis: 0 for no staining; 1 for basal layer staining; 2 for basal and parabasal layer staining; and 3 for full thickness staining. In the specimens immunostained for COX-2, the distribution (the percentage of positive cells) and the intensity of staining were assessed semiquantitatively. The scoring criteria were similar to those used for human specimens [15]. Both the number of positive cells and the intensity of staining were evaluated as follows. Number of positive cells: none, 0; focal (one third of cells stained), 1; multifocal (two third of cells stained), 2; and diffuse (most cells stained), 3. Intensity of staining: none, 0; mild (between 0 and 2), 1; and strong (clearly identified by × 40 magnification), 2. The scores for distribution and intensity were added. The score for each specimen was then recorded as the mean overall score of five × 40 objective fields. All samples were independently scored by two blinded investigators.

### Chemopreventive agents

Wistar rats (6–7 weeks of age, 200 ± 10 g body weight) were purchased from the animal center of Hubei Chinese Medical College. The animals were housed in plastic cages under standard laboratory condition (25°C; 40% relative humidity; 12 h dark/light cycle) with food and water *ad libitum *for 1 week. After the acclimation period, they were randomly allocated to 5 groups: (A) aspirin, 0.5 g/kg diet, 10 rats; (B) PEITC, 0.5 g/kg diet, 10 rats; (C) aspirin, 0.5 g/kg and PEITC, 0.5 g/kg diet, 10 rats; (D) positive control, 8 rats; (E) negative control, 9 rats. Each rat in groups A-D was given a single dose of NNK (25 mg/kg body weight, intratracheal instillation). Rats in group E were instilled with 0.1 ml iodized oil without NNK. Six hours later, the rats in groups A, B and C were fed their special diets, while groups D and E were maintained on the normal diet as before. The normal diet was produced by the Laboratory of Wuhan University on the basis of rat formula feeds, which are regulated by standard GB 14924-2001 published by the People's Republic of China. The dietary treatments were continued until the end of the experimental period. The rats were weighed weekly and food consumption was measured every other day. At 3 months (91 days) after NNK administration, the rats were killed and the lungs were processed for histopathological and immunohistochemical examination.

### Statistics

Analyses were done using SPSS software version 13.0. One-way ANOVA followed by Dunnett's T3 test was used to evaluate the changes of PCNA protein expression among the groups. One-way ANOVA followed by Tukey's test was used to evaluate changes in COX-2 protein expression, food consumption and body weight among the groups. The incidences of alveolar hyperplasia and dysplasia were compared among the chemopreventive experiment groups using the χ^2 ^test.

## Results

### Instillation region

**Figure 1:**
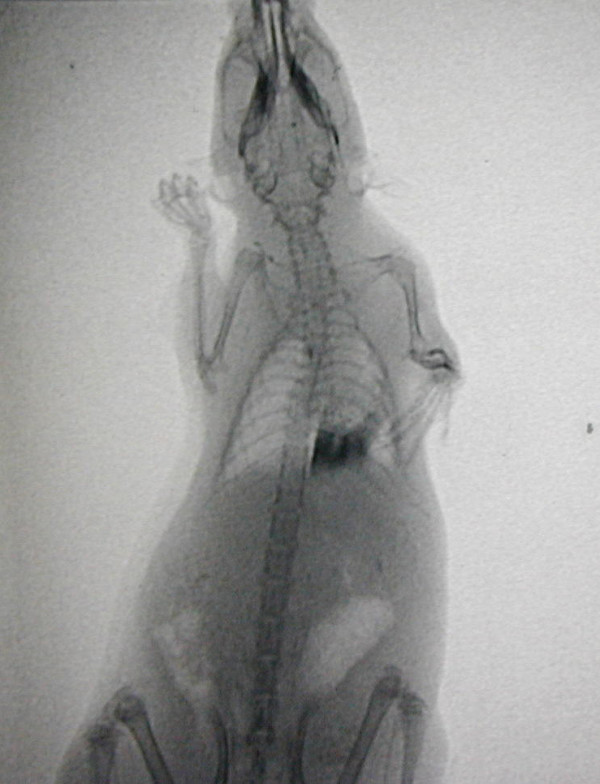
**X-ray photographs of NNK-treated rats**. X-ray photographs showed the carcinogenic iodized oil located in the left lobe after instillation.

All animal pulmograms showed that the iodized oil was located in the left inferior lobe (Fig. 1), and the image was gradually attenuated with time. Three months (92 days) after instillation, the image had almost disappeared.

### Morphological changes

**Figure 2:**
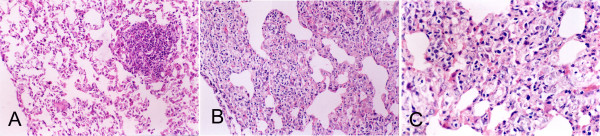
**Photomicrographs of typical lung lesions in NNK-treated Wistar rats**. (A) Alveolar epithelial hyperplasia in a Wistar rat (magnification × 200). (B) Dysplasia occurred in the alveolar region. Some of the alveolar structure was absent and replaced by proliferated cells (magnification × 200). (C) Proliferated cells with increased cellular atypia (magnification × 400).

Group 1 was killed on day 92. In this group, 10 rats (55.56%) were seen by the naked eye to have firm gray nodules in the left inferior lobe, ranging in size from about 2 to 5 mm; 2 rats had pulmonary emphysema; the others had no obvious changes. Microscopy revealed that 6 rats (33.33%) had alveolar hyperplasia: the alveolar structure of lung was present; the proliferated cells obliterated the normal alveolar space but had no obvious cytological atypia. Ten rats (55.56%) showed alveolar atypical dysplasia: the alveolar structure of lung was largely present; the proliferated cells were larger and showed enlarged nuclei, hyperchromasia and nuclear-cytoplasmic reversal (Fig. 2). Only 2 out of 18 rats had squamous metaplasia of the bronchial epithelium, and none had dysplasia or CIS. No pathological changes appeared in any of the control groups.

### Origin of proliferated cells in the NNK-treated group

**Figure 3:**
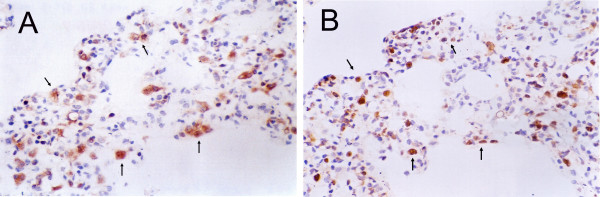
**CK and PCNA expression in alveolar septa of Wistar rats exposed to NNK**. Parallel sections of thickened alveolar septa were stained with CK antibody (A) and PCNA-specific antibody (B). Arrows indicate cells that expressed both CK and PCNA. Magnification × 400.

**Figure 4:**
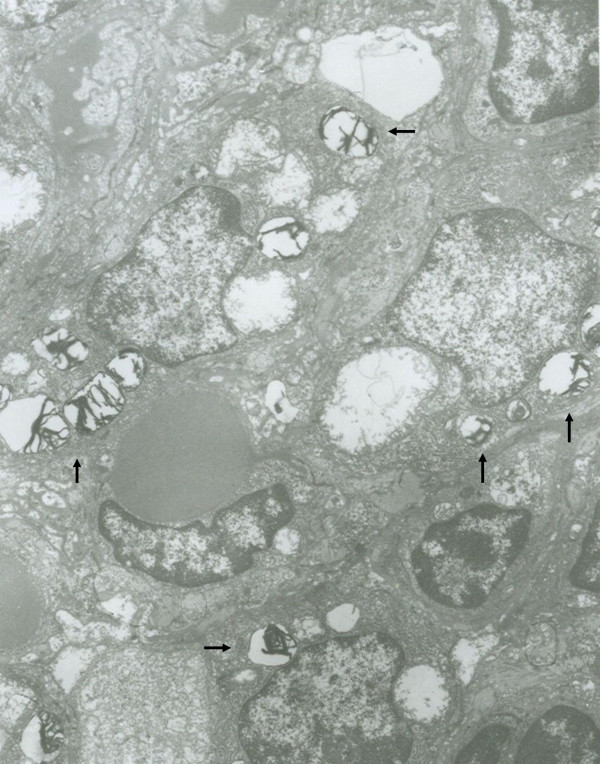
**Transmission electron microscopy of proliferated cells in Wistar rats exposed to NNK**. The proliferated cells had lamellar body-like structures (arrows). Magnification × 4000.

In order to determine the origin of the proliferated cells, we studied CK and S-100 expression in lung specimens. CK was positive in some proliferated cells, but S-100 was negative. In the thickened alveolar septa, some cells expressed both CK and PCNA (Fig. 3A, B). By transmission electron microscopy, the proliferated cells had lamellar body-like structures; the mitochondria in these cells were swollen (Fig. 4).

### COX-2, p63 and PCNA expression in preneoplastic lesions in the NNK-treated rats

**Figure 5:**
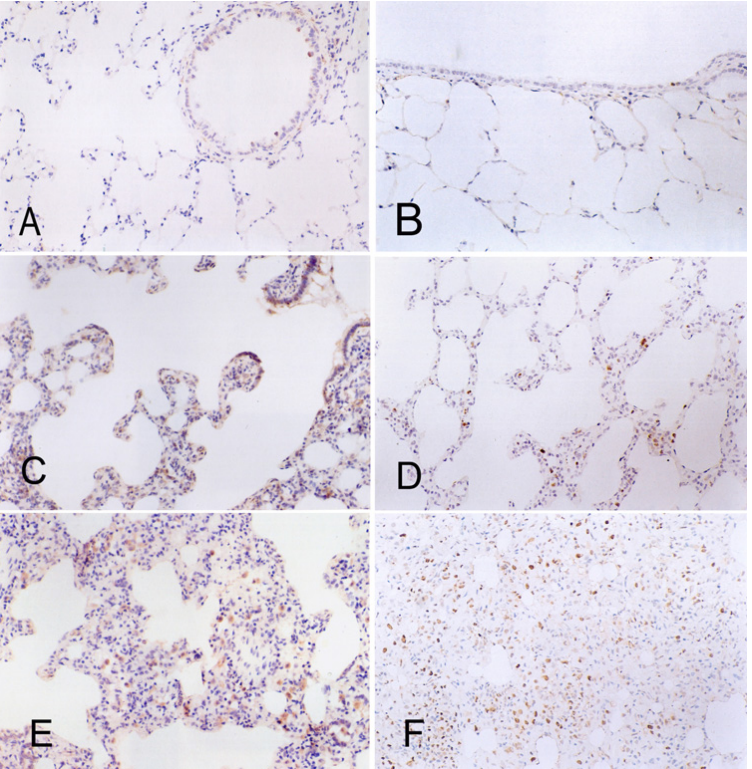
**Expression of COX-2 and PCNA in preneoplastic lung lesions of Wistar rats exposed to NNK**. A and B show immunohistochemical staining for COX-2 and PCNA in normal lung tissues of rats. A, COX-2; B, PCNA. C and D show immunohistochemical staining for COX-2 and PCNA in alveolar hyperplasia of NNK-treated Wistar rats. C, COX-2; D, PCNA. E and F show immunohistochemical staining for COX-2 and PCNA in alveolar dysplasia of NNK-treated Wistar rats. E, COX-2; F, PCNA. Magnification × 200.

In normal rat lung tissue, bronchial mucosa epithelial cells and smooth muscle cells stained positively for COX-2. Some basal cells in normal bronchial epithelium expressed p63 and PCNA. In the control group only 2 specimens showed no reaction for p63, the mean (SD) scores for COX-2 and PCNA are 0.68 (0.33) and 1.16 (0.53), respectively. In preneoplastic lesions in the NNK-treated group, the mean (SD) scores for COX-2 were 2.93 (0.24) and 3.34 (0.43) in alveolar hyperplasia and alveolar atypical dysplasia, respectively. p63 protein was not expressed anywhere in these specimens except in the bronchial mucosa. The mean (SD) PCNA indices were 18.57 (2.60) and 24.25 (3.34) in alveolar hyperplasia and alveolar atypical dysplasia, respectively. Compared with normal tissues, COX-2 expression also clearly increased in alveolar hyperplasia, but there was no difference between alveolar hyperplasia and alveolar atypical dysplasia. The PCNA labeling index increased in cases of alveolar hyperplasia, and a significant difference was detected between alveolar hyperplasia and alveolar atypical dysplasia (Fig. 5).

### Chemoprevention

**Figure 6:**
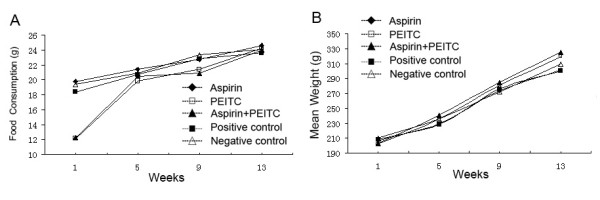
**Food consumption and body weight in groups A-E**. A, food consumption was measured every other day and averaged for all rats in the group; B, body weights were measured weekly and averaged for all rats in the group. Values shown are means.

**Figure 7:**
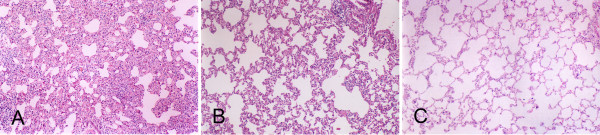
**Aspirin and/or PEITC inhibit lung tumorigenesis in Wistar rats after NNK instillation**. Treatment with aspirin and/or PEITC reduced alveolar dysplasia and improved lung structure after 91 days. A, NNK-treated group; B, aspirin group; C, aspirin and PEITC group. Magnification × 100.

In the second part of our study, we explored the ability of PEITC and aspirin to inhibit the effect of NNK in the Wistar rat model of preneoplastic lesions. Figure 6 presents the food consumption and body weight throughout the experiment. Food consumption was lower in groups B (PEITC) and C (aspirin and PEITC) than in the other groups during week 1 (P < 0.01), especially on the second day, and lower than in group E (negative control) during week 9 (P < 0.05). There were no effects on body weight. In the carcinogen-treated control group (group D), the incidences of alveolar hyperplasia and alveolar atypical dysplasia were 25.00% and 62.50% respectively. Progression to adenocarcinoma was inhibited in groups A, B and C. Treatment with aspirin and/or PEITC reduced the alveolar dysplasia and improved the lung structure (Fig. 7). The results are summarized in Table 1. The incidence of atypical dysplasia was significantly lower in groups A (aspirin), B (PEITC) and C (aspirin and PEITC) than in the carcinogen controls (group D). In group A, aspirin significantly (P < 0.01) decreased atypical dysplasia by 84.00%. In group B, PEITC also significantly (P < 0.01) decreased atypical dysplasia by 84.00%. In group C, atypical dysplasia was reduced by 100.00%. Alveolar hyperplasia was also reduced in group C but the reduction was not statistically significant. No alveolar hyperplasia or atypical alveolar dysplasia appeared in any of the negative controls, which were not treated with NNK (group E).

**Table 1:** The effects of aspirin or/and PEITC on lung tumorigenesis in Wistar rats treated with NNK

Group	Number of rat	Alveolar hyperplasia		Alveolar atypical dysplasia	
		
		n	Incidence (%)	n	Incidence (%) [Decrease^a^]
A. Aspirin	10	6	60.00	1	10.00^◆^ [84.00%]
B. PEITC	10	4	40.00	1	10.00^◆^ [84.00%]
C. Aspirin + PEITC	10	1	10.00	0	0.00^◆◆^ [100.00%]
D. Positive control	8	2	25.00*	5	62.50**
E. Negative control	9	0	0	0	0

^a ^Compared with the incidence of alveolar atypical dysplasia in group D

*p < 0.05 when compared with negative control group by Fisher's exact probability test.

**p < 0.01 when compared with negative control group by Fisher's exact probability test.

^◆^p < 0.05 when compared with positive control group by Fisher's exact probability test.

^◆◆^p < 0.01 when compared with positive control group by Fisher's exact probability test.

The specimens were further analyzed for proliferation and expression of COX-2 to elucidate the mechanism by which aspirin or/and PEITC reduced alveolar atypical dysplasia. The data presented in Table 2 summarize the PCNA labeling indices and COX-2 scores for each group. The percentage of PCNA positive cells was significantly reduced by aspirin and PEITC in group C, and also reduced in groups A and B by aspirin or PEITC. However, the reduction was not statistically significant. COX-2 expression was not changed by PEITC treatment; in contrast, it was clearly reduced by aspirin or by aspirin and PEITC.

**Table 2:** The effects of aspirin or/and PEITC on the expressions of PCNA and COX-2

Group	Number of rat	% Cells Positive for PCNA Mean (SD)	Scores for COX-2 Mean (SD)
A.aspirin	10	12.05(8.81)	2.08(0.72)^◆^
B.PEITC	10	10.92(11.55)	2.34(0.95)
C.asprin + PEITC	10	4.25(5.27)^◆◆^	1.60(0.73)^◆◆^
D.Positive control	8	24.48(9.14) **	3.13(0.88) **
E.Negative control	9	1.17(0.56)	0.67(0.35)

*p < 0.05 when compared with negative control group.

**p < 0.01 when compared with negative control group.

^◆ ^p < 0.05 when compared with positive control group.

^◆◆^p < 0.01 when compared with positive control group.

## Discussion

The Wistar rat has been used widely in experimental research. Unlike the A/J mouse, it is not susceptible to the development of adenomas that progress with time to carcinomas. In 1984, we successfully established a protocol for inducing squamous cell carcinoma in Wistar rat lungs by MCA [12]. In this model, we could obtain different morphological stages in the mutistep process of lung tumorigenesis by controlling the survival time: hyperplasia, squamous metaplasia, atypical dysplasia, CIS, invasive carcinoma and metastatic carcinoma. Using this model, we explored many aspects of the molecular mechanism of formation of pulmonary squamous cell carcinoma. This study demonstrated for the first time that a single-dose intratracheal instillation of NNK could produce preneoplastic lesions in Wistar rat lungs. Three months after instillation, the rats treated with NNK had no obviously pathological lesions in any of the main organs except the lung. First, this protocol has the advantage of being safe, since the carcinogens are handled only once. To our knowledge, only one other single dose protocol has been reported to induce adenomas in A/J mice by the i.p. route: Hecht developed a rapid single-dose protocol for inducing lung tumors in A/J mice by NNK in 1989. A single intraperitoneal injection of NNK at a dose of 2 mg per mouse was sufficient to induce an average of 10–12 lung adenomas in each animal [16]. Various investigators have used this model to demonstrate the efficacies of chemopreventive agents. Second, our model is suitable for investigating somatic changes during lung tumorigenesis, including changes in the blood. Although the organospecificity of NNK for the lung is remarkable, most studies have involved systemic administration of the compound, e.g. orally or by i.p. or s.c. injection, so tumors were also found in other organs [17-20]. This may make it difficult for researchers to distinguish specific changes in lung tumorigenesis-related serum proteins. In our study, NNK was administered locally into the respiratory tract. Lesions were found only locally in the lung. The model could be used to identify prognostic and monitoring factors from blood samples for early detection. Third, intratracheal instillation is more relevant to inhalation of tobacco smoke by people. The results show that alveolar hyperplasia and atypical dysplasia can be observed in this model. Some proliferated cells in these lesions had abundant cytoplasm and usually expressed CK abundantly; electron microscopy showed that most of these cells had lamellar body-like structures. This suggests that the proliferated cells may derive from type 2 pneumocytes. According to the dosage reported by Schuller [21], we tested the carcinogenic potency of NNK in preliminary studies on Wistar rats in doses ranging from 25–50 mg/kg body weight over 3 to 6 months, but no dose or time response was observed. Long-term studies will be carried out to test our expectation that the preneoplastic lesions described here will in fact progress to adenocarcinomas. With further development, this should furnish an excellent model for preneoplastic lesions, carcinomas and metastatic carcinomas.

Comparing these two models morphologically, the MCA-induced lung preneoplastic lesions were squamous metaplasia, squamous atypical dysplasia and CIS. The NNK-induced preneoplastic lung lesions were alveolar hyperplasia and atypical dysplasia. This indicates that MCA and NNK have different target locations in lung tumorigenesis.

Carcinogens such as NNK require metabolic activation to exert their carcinogenic effects; there are competing detoxification pathways, and the balance between metabolic activation and detoxification differs among individuals and will affect cancer risk. Prostaglandin (PG)-endoperoxide synthase (cyclooxygenase) enzymes (COX) may play important roles in the oxidation of NNK in lung, and they also catalyze the conversion of certain polynuclear aromatic hydrocarbons and/or metabolites to the ultimately carcinogenic diol epoxides [22,23]. Two isoforms of COX, COX-1 and COX-2, have been identified as the enzymes responsible for the production of prostaglandins. COX-2 increases in many neoplastic tissues and is inducible by cytokines, growth factors and chemical carcinogens. Multiple lines of evidence suggest that COX-2 is also important in carcinogenesis. El-Bayoumy et al. [24] observed that increased COX-2 expression correlated with the development of lung tumors induced by NNK. In humans, overexpression of COX-2 has also been associated with lung cancer, specifically with adenocarcinomas [25,26]. Hida et al. [25] reported that COX-2 was overexpressed in 16 of 23 adenocarcinomas (70%) and one of three AAH. Increased COX-2 expression in precursor lesions of human lung adenocarcinoma was also observed by Yukio et al. [27]. This supports a role for COX-2 in the earliest stages of lung adenocarcinoma progression. Our data provide further confirmation of this role. COX-2 protein levels clearly increased in alveolar hyperplasia and alveolar atypical dysplasia, and the COX-2 score correlated with the PCNA labeling index. COX-2 expression in squamous cell carcinoma is very different from that in lung adenocarcinoma, studies have demonstrated that COX-2 protein and mRNA expression was significantly lower in squamous cell carcinoma than in adenocarcinoma [26,28-30].

The recently discovered factor p63 is the most ancient member of the p53 family [31]. p63 amplification or overexpression is often found in NSCLC, specifically in squamous cell carcinomas [32-34]. To study the role of p63 in tumorigenesis, we analyzed the frequency and timing of p63 protein expression by IHC on NNK-induced lesions. We found that p63 was not overexpressed in any lesion induced by NNK. Conflicting data have been published regarding the immunoreactivity of p63 in AAH [35], indicating a need for further investigation.

Aspirin is a traditional non-steroidal anti-inflammatory drug (NSAID). There is some evidence that regular and prolonged aspirin use is associated with a reduced risk of lung cancer [36-41]. One proposed mechanism for the chemopreventive properties of aspirin and other NSAIDs entails the inhibition of COX-2 [42], the enzyme that regulates prostaglandin production. Prostaglandin E_2 _plays a key role in the accelerated proliferation of lung cancer cell lines [43]. The results from the first part of our study suggested that COX-2 is important in NNK-induced tumorigenesis. So, in the second part of the study, we used the NNK model to observe the chemopreventive efficacy of aspirin via COX-2 suppression. Our results confirmed that 25 mg/kg body weight aspirin per day significantly reduced COX-2 expression in lung, and also lowered the incidence of alveolar dysplasia. However, aspirin failed to decrease the proliferation of the alveolar epithelium. This suggests that increased levels of COX-2 do not directly lead to proliferation in the early stage of NNK-induced tumorigenesis. Duan [44] declared that aspirin was not able to inhibit A549 cell proliferation directly without cytokine stimulation. In 2004, Keith et al. [45] found that increased pulmonary production of prostaglandin I2 (prostacyclin) by lung-specific overexpression of prostacyclin synthase decreases lung tumor incidence and multiplicity in chemically-induced murine lung cancer models. Hence, the modulation of pathways downstream of COX-2 may be well worth exploring in lung cancer.

PEITC, a naturally occurring constituent of cruciferous vegetables, has been reported to inhibit the development of tobacco-specific carcinogen-induced lung tumors [46-50]. Morse et al. [46] reported that F344 rats fed diets containing PEITC (3 μmol/g diet), before and during treatment with the NNK, developed about 50% fewer lung tumors than NNK-treated rats fed control diets. The lung tumor incidences in the NNK-treated groups, fed a diet containing 4 mmol/kg or 8 mmol/kg PEITC, were 9% and 17% respectively, compared to 67% in the control group [49]. In this study, we observed inhibition of lung tumorigenesis in Wistar rats fed with PEITC after NNK instillation. PEITC significantly lowered the incidence of alveolar dysplasia in group B, but we observed no down-regulation of COX-2 expression or PCNA in this group. The anticarcinogenic action of isothiocyanates against lung cancer has been attributed to the inhibition of phase I enzymes and/or to the induction of activity of phase II conjugation enzymes for carcinogen metabolism [51-54]. In most reported studies, PEITC must be present at the time of carcinogen exposure in order to inhibit tumorigenesis detectably [48,55,56]. The results indicate that PEITC inhibits the progression to alveolar dysplasia in early stages of tumorigenesis, and the inhibitory effect may be COX-2 independent. However, a previous study concluded that PEITC decreased COX-2 protein expression levels, leading to reduced secretion of both pro-inflammatory mediators in Raw 264.7 macrophages [57]. The environment is very complex *in vitro*; many factors participate in tumorigenesis. In cell culture, COX-2 expression has proved to be highly responsive, changing rapidly in the presence of many growth factors, cytokines and other inflammatory mediators. It is therefore not surprising that COX-2 expression becomes up-regulated in some circumstances. Despite strong evidence that PEITC inhibits cancer development, there are also reports that it induces or promotes carcinogenesis. The molecular basis of the latter may be PEITC-increased transactivation of activator protein 1 (AP-1) and AP-1 DNA binding [58]. In contrast, some studies have reported that aspirin exerts antitumor effects partly through blocking the induction of activator protein-1 (AP-1) activation by tumor promoters [59]. Our results showed that a combination of aspirin and PEITC reduced proliferation significantly. This could partly explain why there was significant reduction of PCNA expression in group C.

Lung tumorigenesis is a complex process in which many factors participate. These finding are important for future research into lung cancer chemoprevention and therapy in smokers and ex-smokers with early lesions.

## Conclusion

A single-dose of NNK at 25 mg/kg body weight by intratracheal instillation is sufficient to induce preneoplastic lesions in Wistar rats. COX-2 takes part in NNK-induced tumorigenesis, but a decrease in this enzyme fails to inhibit proliferation of the alveolar epithelium. Aspirin and PEITC inhibit the progression of NNK-induced preneoplastic lesions by reducing cellular proliferation.

## Competing interests

The author(s) declare that they have no competing interests.

## Authors' contributions

FY, HLC and DX participated in the study design and manuscript preparation. BY and YXZ participated in the study design, statistical analysis, and manuscript preparation and drafting. MQL and BTL conceived the study and participated in its design and manuscript preparation.

## Pre-publication history

The pre-publication history for this paper can be accessed here:


